# Auer Rod in a Neutrophil in a Nonmalignant Condition

**DOI:** 10.4274/tjh.2015.0275

**Published:** 2016-05-16

**Authors:** Harish Chandra, Smita Chandra, Vibha Gupta, Divyaa Mahajan

**Affiliations:** 1 Himalayan Institute of Medical Sciences, Department of Pathology, Dehradun, India

**Keywords:** Auer rods, Neutrophil, Typhoid fever

Auer rods are normally observed in immature myeloid precursors including myeloblasts and promyelocytes in cases of acute myeloid leukemia, while cases have rarely reported Auer rods in polymorphs in acute myeloid leukemia [1,2].

A 19-year-old female presented with high-grade fever and abdominal pain for 1 week. Her laboratory investigations revealed hemoglobin of 65 g/L, red blood cell count of 3.3x1012/L, mean cell volume of 96.2 fL, white blood cell count of 8.5x109/L, and platelet count of 23x109/L. She was found to be positive for Salmonella Typhi antigen O in 1:160 dilutions (slide agglutination by Beacon Diagnostics, India). An interesting finding was observed during her peripheral blood examination, which showed an Auer rod-like structure within the cytoplasm of a neutrophil, along with features of dysmyelopoiesis (Figure 1). Bone marrow aspiration was done, which was unremarkable and showed normoblastic maturation.

To the best of our knowledge, no case has been reported in the literature with Auer rods in a nonmalignant condition. Therefore, the present case is being reported, which shows an Auer rod in a polymorph in a case of typhoid fever.

## Ethics

Informed Consent: It was taken.

## Figures and Tables

**Figure 1:**
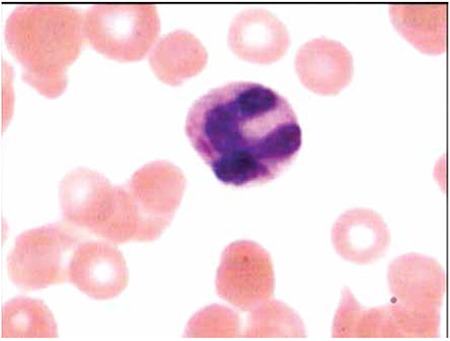
Neutrophil shows an Auer rod at 100x (Jenner-Giemsa stain), 270x203 mm (72x72 dpi).
